# Editorial: Clinical and Hemodynamic Performance of Aortic Endografts

**DOI:** 10.3389/fsurg.2018.00044

**Published:** 2018-06-22

**Authors:** Efstratios Georgakarakos

**Affiliations:** Department of Vascular Surgery, Democritus University of Thrace, University Hospital of Alexandroupolis, Alexandroupolis, Greece

**Keywords:** hemodynamics, endovascular therapy, aneurysm, bioengineering, endografts

Endovascular Aneurysm Repair (EVAR) is considered the treatment of choice in the majority of abdominal aortic aneurysms (AAA) since it is associated with low perioperative morbidity and mortality as well as a shortened hospital stay compared to open surgical repair (1, 2).

At first, classic endograft (EG) design was uniform and simple; it comprised a bifurcated configuration of fabric (either polutetrafluoroethylene of woven polyester) sewn with a self-expanding Nitinol endoskeleton (3). The latter ensured the central and distal sealing by means of stent oversizing whereas the central fixation was enhanced via hooks, pins, barbs, and anchors (4). Both hemodynamic and clinical performance of endografts are significantly affected by several geometric factors, the most important of which are the infrarenal neck angulation, diameter and shape, the curvature of EG, and the ratios of the proximal-to-distal diameters (Georgakarakos et al.) (5, 6).

According to the Human Aortic Anatomy Project, only 32% of men and 12% of women fulfill all 3 instructions for use (IFU) for the infrarenal neck (length, angulation, diameter) for AAA of 5–5.5 cm, whereas these percentages decrease significantly for AAA greater than 6–6.5 cm (7). Notably, EVAR outside-the-IFU is adapted by a significant percentage of physicians in real-world practice, putting those patients in significantly greater risk of developing type-I endoleak with consequent increased aneurysm-related mortality (8, 9). Therefore, EVAR evolution is characterized by significant modifications and developments in the design of aortic endografts in order to enhance the mechanical stability, practical applicability, and clinical endurance in the long term (Scaife et al.; Kontopodis et al.).

Interestingly, the need for minimally invasive repair of endovascular failures (i.e., avoiding the open repair or conversion) led to custom-made designs or EG hybrid solutions that combine components from different commercially available endografts (i.e., main-bodies/aortic cuffs/iliac limbs) with different biomechanical and structural properties (stress on metallic endoskeleton, strains in fabric) (10, 11). Notably, altered geometrical patterns of these new designs, such as reduced mainbody length (i.e., reduced mainbody-to-iliac limbs ratio) differentiate certain hemodynamic indices such as drag forces, shear stress or pressure distribution along the endograft (12, 13). Moreover, emerging endovascular technologies focusing on intrasac sealing rather that infrarenal fixation comprise a totally new research field, since the hemodynamic basis of mid- and long-term sealing are not fully understood (14). Interestingly, novel developments such as pivotal-FEVAR, low-profile endografts to overcome the stenosed and angulated iliac arteries burden as well as endoanchoring to treat migration and type-I endoleaks are worth mentioning innovations, the long-term efficiency of which has to be proven (3, 15). Lastly, the hemodynamic performance of parallel grafts used in complex repair techniques (chimneys, snorkels, and fenestrated EVAR) remains to be investigated in order to gain information to aid the development of dedicated stent-graft types for improvement of the long-term results of the aforementioned techniques (Kandail et al.).

## Author contributions

The author confirms being the sole contributor of this work and approved it for publication.

### Conflict of interest statement

The author declares that the research was conducted in the absence of any commercial or financial relationships that could be construed as a potential conflict of interest.
